# Galectin-3 Promotes Müller Glia Clearance Phagocytosis *via* MERTK and Reduces Harmful Müller Glia Activation in Inherited and Induced Retinal Degeneration

**DOI:** 10.3389/fncel.2022.878260

**Published:** 2022-05-31

**Authors:** Deborah S. Lew, Morgan J. McGrath, Silvia C. Finnemann

**Affiliations:** Center for Cancer, Genetic Diseases and Gene Regulation, Department of Biological Sciences, Fordham University, Bronx, NY, United States

**Keywords:** retina, retinal degeneration, phagocytosis, galectin-3, glia, Müller cells

## Abstract

Clearance phagocytosis is a documented function of Müller glia in the retina. However, the molecular mechanisms of Müller glia phagocytosis remain largely undefined. Here, we show that extracellular galectin-3 and protein S promote clearance phagocytosis by immortalized human MIO-M1 Müller cells in an additive, saturable manner. Galectin-3 promotes phagocytosis by primary Müller glia from wild-type (WT) mice but not from mice that lack the engulfment receptor MERTK and therefore develop postnatal photoreceptor degeneration. Probing a possible functional link between Müller galectin-3 and MERTK, we discovered that *mertk*^−/−^ Müller glia *in situ* show excess galectin-3 at postnatal day 20 (P20), an age prior to detectable photoreceptor degeneration. Moreover, double knockout (DKO) mice lacking both galectin-3 and MERTK show increased activation of Müller cells (but not of microglia) at P20 and more pronounced photoreceptor loss at P35 compared to mice lacking MERTK alone. Exploring the well-established sodium iodate injury model, we also found more severe activation specifically of Müller glia, and worse retinal damage in mice lacking galectin-3 compared to WT mice. Indeed, galectin-3 deficiency significantly increased sensitivity to injury, yielding Müller activation and retinal damage at a sodium iodate concentration that had no effect on the WT retina. Altogether, our results from both inherited and acutely induced models of retinal degeneration agree that eliminating galectin-3 exacerbates Müller cell activation and retinal degeneration. These data identify an important protective role for the MERTK ligand galectin-3 in the retina in restraining Müller glia activation.

## Introduction

Clearance phagocytosis processes are critical to the homeostasis of the healthy vertebrate retina and contribute to retinal degenerative processes. Three professionally phagocytic cell types reside in the mammalian retina, the retinal pigment epithelium (RPE), microglia, and Müller glia. RPE cells, stationary and immortal in the mature retina, are tasked with routine, diurnal phagocytosis of spent photoreceptor outer segment fragments (Young and Bok, 1969). If RPE phagocytosis is impaired to the extent that unengulfed photoreceptor debris builds up in the neural retina, retinal degeneration will result (Dowling and Sidman, 1962). Decades of research have identified a complex phagocytic pathway that depends on the activation of the engulfment receptor MERTK by integrin signaling and by extracellular ligand proteins such as protein S and Gas6 (Finnemann, 2003; Nandrot et al., 2004; Burstyn-Cohen et al., 2012). MERTK, a TAM family receptor tyrosine kinase, is essential to RPE phagocytosis and retinal health, as *mertk* mutations cause *retinitis pigmentosa*-type retinal degeneration in human patients (Gal et al., 2000; Parinot and Nandrot, 2016).

Unlike RPE cells, microglial cells are phagocytic cells that are highly migratory regardless of whether they have infiltrated or are resident of the retina. Phagocytic inclusions in microglia are found in human retinal diseases (Gupta et al., 2003). Microglia inhibition or ablation studies in animal models have shown that microglia activities such as clearance phagocytosis may increase or reduce the severity of retinal diseases (Silverman and Wong, 2018). Indeed, Zhao and colleagues have shown that phagocytosis of living photoreceptors by activated microglia aggravates retinal degeneration (Zhao et al., 2015). Mechanistically, retinal microglia likely employ MERTK-dependent and alternate pathways for phagocytosis, although most mechanistic studies have studied non-retinal microglia (recently reviewed by Butler et al., 2021).

Like RPE cells, Müller glia are stationary in the mature retina and perform diverse functions to maintain retinal homeostasis, including clearance phagocytosis of retinal debris (Reichenbach and Bringmann, 2013; Bejarano-Escobar et al., 2017; Sakami et al., 2019). As part of an inflammatory response to retinal damage, Müller cells become activated. In mammalian models, hallmarks of Müller cell activation include increased expression of intermediate filaments including glial fibrillary acidic protein (GFAP) (Bringmann et al., 2009). The phagocytic pathway of Müller glia and its regulation are not yet completely understood. However, the involvement of phosphatidylserine on apoptotic particles and the F-actin regulator Rac1 in Müller glia imply significant similarity to the MERTK-dependent phagocytic pathway of the RPE (Mao and Finnemann, 2012; Ruggiero et al., 2012; Nomura-Komoike et al., 2020).

Galectin-3 is a member of the galectin family of beta-galactoside-binding proteins that has roles in a multitude of different functions, pathways, and pathologies, either as cytosolic or as extracellular protein (recently reviewed by Johannes et al., 2018; Soares et al., 2021). Galectin-3 localization to F-actin-rich phagocytic cups and phagosomes in macrophages suggests an intracellular role in phagocytic pathways (Sano et al., 2003). In contrast, extracellular galectin-3 promotes apoptotic cell clearance phagocytosis by hepatic stellate cells (Jiang et al., 2012). Cell culture experiments have further demonstrated that extracellular galectin-3 may bind to MERTK to stimulate apoptotic debris clearance (Caberoy et al., 2012). Characterization of galectin-3 in the retina is still incomplete. With respect to retinal expression, RPE and Müller glia in pig and mouse retina express galectin-3 constitutively (Kim et al., 2009; Esposito et al., 2021), and retinal microglia upregulate galectin-3 upon activation (Bauer et al., 2016). With respect to retinal physiology, galectin-3 has been shown to serve as a neuroprotectant or enhance retinal pathology. With respect to contribution to specific retinal cell-molecular pathways, we previously found no role for galectin-3 in routine diurnal clearance phagocytosis by RPE cells either *in vivo* or in cell culture, and *lgals3*^−/−^ mice show no obvious retinal functional or morphological abnormalities (Esposito et al., 2021). The specific roles of galectin-3 in activated microglia and Müller glia have not yet been fully elucidated. Finally, it is not fully understood if galectin-3 expression results from glia activation or if it contributes to the activation mechanism itself.

In this study, we first explored Müller cells in cell culture clearance phagocytosis assays. The results indicated that Müller cells employ extracellular galectin-3 as an MERTK ligand for phagocytosis. These findings prompted us to investigate galectin-3 in two distinct, well-established *in vivo* animal models of photoreceptor degeneration, due to lack of MERTK and sodium iodate chemical injury, respectively. Altogether, these *in vivo* results reveal a novel protective role for galectin-3 in curbing retinal damage associated with excess Müller glia activation.

## Materials and Methods

### Reagents

All reagents were from Millipore-Sigma (St. Louis, MO) or Thermofisher (Carlsbad, CA) unless otherwise indicated.

### Cell Culture

The human Müller cell line Moorfields/Institute of Ophthalmology-Müller 1 (MIO-M1) was obtained from the UCL Institute of Ophthalmology, London, UK. They were extensively characterized earlier (Limb et al., 2002). Cells were maintained in high glucose DMEM with 10% fetal bovine serum. Cells were seeded at ~50% confluence into 96-well plates with and without coverslips as appropriate and maintained for 3–5 days before phagocytosis experiments. Primary Müller cells were isolated from neural retinas from 2 to 4-week-old mice, with retinas from seven mice pooled to yield one primary cell culture using a papain-based tissue dissociation system according to the manufacturer’s instructions (#LK003150, Worthington Biochemical Corp, Lakewood, NJ). In brief, retinas were dissociated with papain for 1 h at 37°C. Pelleted cells were resuspended, and live cells were separated from debris using an ovomucoid protease inhibitor gradient before being plated on tissue culture plastic coated with 0.1% gelatin. Primary Müller cells were maintained in high glucose DMEM with 10% fetal bovine serum with medium changes every 7 days, and the cells were passaged after reaching confluence two times to eliminate contaminating cell types. For phagocytosis experiments, passage two primary mouse Müller cells were seeded at ~50% confluence onto gelatin-coated glass coverslips and used for experiments 2–4 weeks later.

### Phagocytosis Assays

Photoreceptor outer segment fragments (POS) mimicking apoptotic debris were used as phagocytic particles in cell culture phagocytosis assays (Finnemann and Rodriguez-Boulan, 1999). POS was purified from pig eyes according to well-established published protocols (Parinot et al., 2014), FITC-labeled immediately prior to use by incubation on a rotator for 1.15 h at room temperature with 0.01 mg/ml FITC isomer-I in 0.1 M Na-bicarbonate buffer, pH 9.5, and washed three times in serum-free DMEM. Müller cells in culture were pretreated with serum-free DMEM for 30 min followed by challenge with FITC-labeled POS in serum-free DMEM with or without recombinant human protein S (Aniara, West Chester, OH) or human or mouse galectin-3 (R and D Systems, Minneapolis, MN) at 0.4 or 2 μg/ml each, or a mix of both ligands at either concentration. Müller cells were incubated for 3 h (MIO-M1 cells) or 5 h (mouse primary Müller cells) before harvest for lysis and immunoblotting or fixation for fluorescence microscopy. To prepare samples for lysis, cells were washed once with PBS, incubated with PBS supplemented with 2 mM EDTA for 10 min to remove surface-bound POS, and washed two more times with PBS. To prepare samples for microscopy, cells were washed three times with PBS prior to fixation with 4% PFA in PBS followed by permeabilization with 0.5% Triton-X100 in OPBS for 15 min and DNA labeling with DAPI at 0.1 μg/ml in PBS for 10 min followed by for fluorescence microscopy of POS and nuclei counterstain. F-actin in PFA-fixed, permeabilized MIO-M1 cells were stained with AlexaFluor594-labeled phalloidin at 1 unit/ml in PBS for 1 h prior to DAPI staining.

### Animals and Animal Treatment

Mice were housed in a 12-h light/12-h dark light cycle with standard food and water *ad libitum*. Cohorts used for experiments were a mix of male and female mice. 129T2/SvEmsJ mice were studied as wild-type (WT) strains, and mutant strains were extensively and routinely backcrossed with WT to maintain 129T2/SvEmsJgenetic background. We have previously characterized *Mertk*^−/−^ and *lgals3*^−/−^ in this background (Mazzoni et al., 2019; Esposito et al., 2021). We crossed these strains to yield *mertk*^−/−^/*lgals3*^−/−^ double knockout mice, from hereon termed DKO mice (Supplementary Figure S1). Like *mertk*^−/−^ and *lgals3*^−/−^ single mutant mice, DKO mice exhibited no overt abnormality with respect to gross morphology, behaviors, body weight, or breeding up to 9 months of age, the latest age studied. For experiments, age-matched cohorts of mice were raised and sacrificed at defined ages as stated in the text and legends for individual experiments. To induce retinal degeneration, sodium iodate freshly dissolved in PBS was injected at 50 mg/kg or 30 mg/kg intraperitoneally in mice on postnatal day 28 (P28; Chowers et al., 2017). Sibling control mice received PBS injections. Analyses were performed with investigators blind to animal treatment. Animal sacrifice and tissue harvest followed 5 days after injections. Mice were euthanized by CO_2_ asphyxiation before immediate eye enucleation and processing. All tissue harvest was done at 3–4 h after light onset to avoid variability due to circadian or diurnal effects. Eyeballs were chilled in HBSS, dissected, and neural retina tissue fractions flash-frozen for immunoblotting. For tissue sectioning, the cornea and lens were dissected prior to immersion fixation of tissue in 4% paraformaldehyde in PBS for 30 min followed by sequential dehydration and embedding in paraffin.

### Tissue Staining and Microscopy Analysis

Seven micrometer thick microtome sections cut at a distance of ~200 μm from the optic nerve were deparaffinized. Epitope unmasking was performed by boiling for 10 min in 10 mM citric acid, 0.05% Tween-20, pH 6. Sections were then blocked with PBS, 1% BSA, and 0.01% Triton-X100 and incubated sequentially with primary and appropriate AlexaFluor-conjugated secondary antibodies. For co-staining of glutamine synthetase and galectin-3, frozen sections of wild-type mouse eyeballs were generated and stained according to standard protocols described previously. Primary antibodies used are listed in Supplementary Table S1. DAPI was used to counterstain nuclei.

Vectashield-mounted samples were imaged using a Leica TSP8 laser scanning confocal microscopy system. X-Y stacks were collapsed to yield maximal projections representing equal tissue volume for image quantification.

Quantification of photoreceptor nuclei rows in tissue sections was performed as in our earlier studies (Lew et al., 2020). Image quantification was performed manually aided by ImageJ software. In each section, the number of photoreceptor rows was counted in three regions of the image (left, central, and right) and averaged. Three retinas from three different mice were analyzed for each sample type.

### SDS-PAGE and Immunoblotting

Cells or dissected tissues were lysed in HNTG buffer (50 mM HEPES, 150 mM NaCl, 1% Triton-X100, 10% glycerol, pH 7.5) freshly supplemented with a protease inhibitor cocktail. Proteins from cleared lysates were separated by SDS-PAGE on 4%–20% gradient polyacrylamide gels (Biorad, Hercules, CA) and transferred to nitrocellulose membranes. Blots were blocked in 10% non-fat milk powder in TBS before incubation with primary and appropriate HRP-conjugated secondary antibodies (Kindle Biosciences, Greenwich, CT), and enhanced chemiluminescence digital detection using a KwikQuant Imager (Kindle Biosciences). Band densities were quantified using the KwikQuant imaging software. Primary antibodies used are listed in Supplementary Table S1.

### Statistical Analysis

All data were collected from at least three independent experiments with biological replicates in each experiment. Single data points were obtained from tissue samples from different mice; tissues were not pooled for immunoblotting. For histology, one eye of a given mouse was evaluated as a single data point. The two eyes from each experimental animal were processed separately and for different applications; i.e., one eye was dissected for lysis and immunoblotting, and the other eye was fixed and processed for histology. The means and standard deviations were calculated for each dataset comprised of data points (n) from individual tissues. Comparisons between two groups were performed using the Student’s two-tailed *t*-test. Comparisons between three or more groups were performed using one-way or two-way ANOVA as appropriate, with Tukey’s *post-hoc* test for comparison of two groups within multiple groups. *P-*values below 0.05 were considered statistically significant for all experiments.

## Results

### Human and Murine Müller Glia Cells in Culture Employ Extracellular Galectin-3 for Clearance Phagocytosis

**Figure 1:**
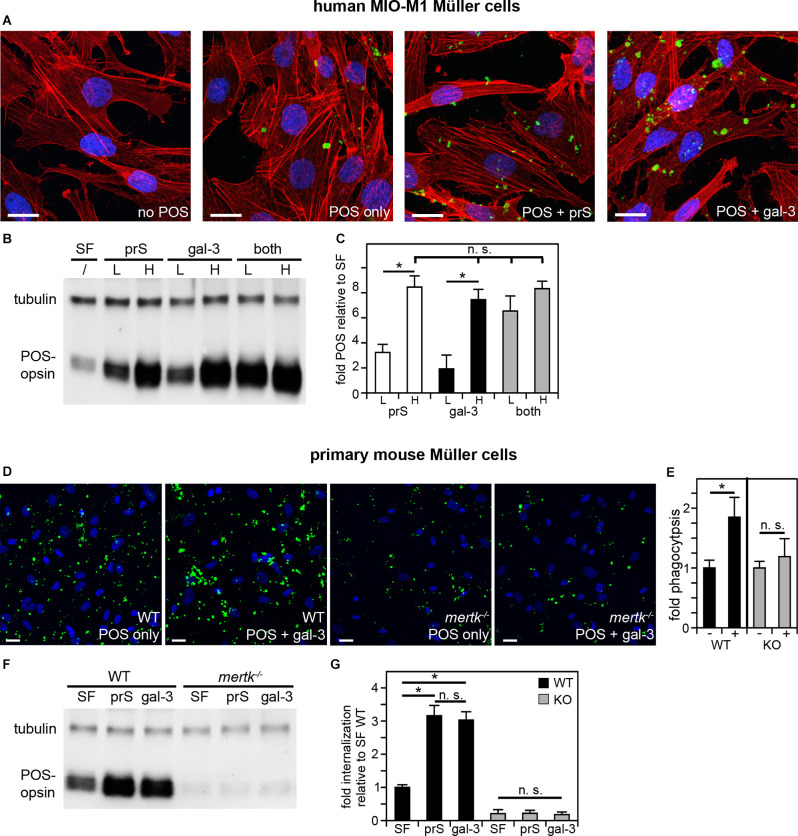
Müller cells in culture phagocytose in a MERTK-dependent manner using either protein S or galectin-3 as MERTK ligands. POS phagocytosis assays explored immortalized human MIO-M1 Müller cells **(A–C)** or primary mouse Müller cells **(D–G)** in culture followed by immunofluorescence microscopy or lysis and immunoblotting. **(A–C)** Human MIO-M1 Müller cells were challenged with fluorescent POS in serum-free medium with or without added MERTK ligands at high concentration (2 μg/ml) for microscopy **(A)** or at high concentration (H) or low concentration (L, 0.4 μg.ml) as indicated for immunoblotting **(B,C)**. Combination treatments were a mix of both ligands with both at low or high concentration. Representative maximal projections in **(A)** show POS (green) and F-actin (red) and nuclei (blue). Scale bars: 20 μm. A representative immunoblot membrane is shown in **(B)** directly comparing POS uptake indicated by levels of the POS marker protein rhodopsin relative to tubulin, which was used as sample loading control. Bars in **(C)** show quantification of blot results as in **(B)** to directly compare levels of phagocytosed POS in each condition. Bars show mean ± s. d.; *n* = 4 independent experiments. ANOVA *post-hoc* test revealed differences as indicated (n. s., not significant; *p < 0.05). ANOVA also revealed significant differences (*p* < 0.05) to cells without added ligands for all single and combined ligand conditions; these differences are not indicated in the bar graph to maintain clarity. **(D)** WT or *mertk*^−/−^ (KO) mouse Müller cells in primary culture were challenged with fluorescent POS in serum-free medium with or without added MERTK ligands as indicated followed by fluorescence microscopy analysis. Representative maximal projections in **(D)** show POS (green) and nuclei (blue). Scale bars: 20 μm. Bars in **(E)** show quantification of total POS uptake from data as in **(D)**. Bars show the comparison of cells receiving galectin-3 (+) compared to the same cells receiving POS alone (-), which are set as 1 for each genotype; mean ± s. d.; *n* = 3 independent experiments per genotype. Data were analyzed by cell type using Student’s *t*-test; * indicates *p* < 0.05. Panel **(F)** shows a representative immunoblot membrane of phagocytosis assays [assays performed as in **(D)**] followed by 2 mM EDTA in PBS wash to remove unengulfed, surface-bound POS prior to cell lysis to allow measurement of internalized POS. Blots were probed for the POS marker rhodopsin and for tubulin, which was used as sample loading control. Bars in **(G)** show quantification of blot results as in **(F)** comparing levels of internalized POS normalized to tubulin among samples. Bars show mean ± s. d.; *n* = 3 independent experiments. ANOVA *post-hoc* test revealed significant effects of protein S and galectin-3 on WT Müller cell but not *mertk*^−/−^ Müller cell internalization as indicated; (n. s., not significant; *p < 0.05). Additionally and not indicated in the figure, *mertk*^−/−^ internalization was significantly lower than WT internalization regardless of ligand addition (*p* < 0.05).

Clearance phagocytosis has been demonstrated to be one aspect of Müller glia cell functionality, but the molecular mechanisms involved have not yet been dissected experimentally. Cell culture phagocytosis assays have been proven highly informative for other cell types and, thus far, have proven to share molecular pathways used by said cell type *in situ*. Therefore, we investigated immortalized human MIO-M1 Müller glia cells challenged with purified photoreceptor outer segment particles (POS). POS serve as model particles for efferocytosis-type clearance phagocytosis, as they expose the universal “eat me” signal phosphatidylserine (Ruggiero et al., 2012) and compete with apoptotic cells for recognition and engulfment (Finnemann and Rodriguez-Boulan, 1999). Fluorescence microscopy showed that MIO-M1 Müller glia phagocytosed higher numbers of POS over a 3-h period of incubation when purified ligands for the engulfment receptor MERTK, protein S or galectin-3 were added during POS challenge (Figure 1A). Protein S is a well-characterized ligand for TAM receptors that is known to act as a physical bridge between TAM receptors on engulfing cells and particles externalizing phosphatidylserine, while galectin-3 has been shown to be able to activate MERTK but has not yet been shown to participate in MERTK-dependent physiological functions. Quantification of POS uptake using western blotting following the same phagocytic challenge further revealed that either protein S or galectin-3 increased POS uptake by MIO-M1 Müller cells in a saturable and concentration-dependent manner and that the effects of protein S and galectin-3 were additive at sub-saturating concentrations (Figures 1B,C). In contrast, adding both ligands at saturating concentrations did not further increase POS uptake (Figures 1B,C). Together, these data indicate that protein S and galectin-3 act on the same pathway and share the same target (Figure 1C). Next, we tested POS phagocytosis by primary Müller cells prepared from the mouse retina. Fluorescence microscopy of phagocytosed (bound or engulfed) POS showed that wild-type mouse Müller glia, like human MIO-M1 Müller cells, increased POS uptake in response to galectin-3 (Figures 1D,E). In contrast, Müller glia from MERTK-deficient mice did not respond to either protein (Figures 1D,E). To analyze specifically engulfed POS, we challenged primary Müller cells with POS in the presence or absence of protein S or galectin-3 followed by removal of surface-bound POS using EDTA, lysis, and immunoblotting for the POS marker rhodopsin. These experiments revealed that either protein S or galectin-3 promoted POS internalization by WT Müller cells (Figures 1F,G). In contrast, adding either TAM receptor ligand had no effect on POS internalization by *mertk*^−/−^ Müller cells (Figures 1F,G). Moreover, POS engulfment by *mertk*^−/−^ Müller glia was ~5-fold less than POS engulfment by WT Müller glia in serum-free medium [Figure 1G, compare black (WT) andgray (*mertk*^−/−^) SF bars]. Altogether, these results show that Müller cells in culture, unlike RPE cells, employ extracellular galectin-3 for phagocytosis. Moreover, the results reveal the similarity of human and murine Müller glia phagocytic pathways and dependence on the TAM family engulfment receptor MERTK.

### Increase in Galectin-3 in Müller Cells in MERTK-Deficient Retina Prior to Photoreceptor Degeneration

**Figure 2:**
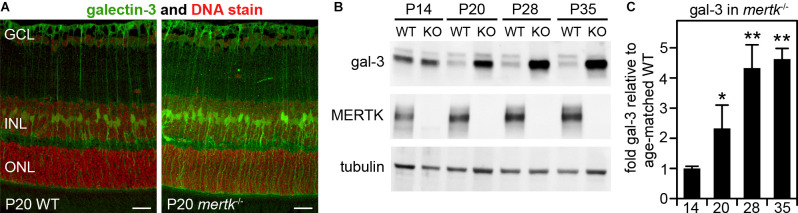
Galectin-3 levels are increased in MERTK-deficient retina as early as P20. Tissues of age-matched WT and *mertk*^−/−^ mice obtained at P20 **(A)** or at ages from P14 to P35 as indicated **(B)** were dissected to yield either embedded samples for sectioning, and subsequent galectin-3 immunofluorescence microscopy **(A)**, or isolated neural retina for immunoblotting **(B,C)**. Representative image panels in **(A)** show galectin-3 (green) and nuclei counterstain (red) in WT (left panel) and *mertk*^−/−^ (right panel) mouse retina cross sections. Retinal layers are marked as follows: GCL, ganglion cell layer; INL, inner nuclear layer; ONL, outer nuclear layer. Scale bars: 20 μm. Representative immunoblots in **(B)** with probes as indicated were quantified to yield differences in galectin-3 levels, which are plotted in **(C)**. Bars show mean ± s. d.; *n* = 3 biological samples from three different mice. Data were analyzed by ANOVA; * indicates *p* < 0.05, ** indicates *p* < 0.01.

In our previous studies focused on galectin-3 in mouse RPE, we noted but did not fully characterize- galectin-3 labeling in WT mouse neural retina (Esposito et al., 2021). We also reported previously that *mertk*^−/−^ mice show no obvious phenotypic abnormality in the retina at P20 but photoreceptor apoptosis by P28 and significant loss of photoreceptors by P35 (Mazzoni et al., 2019). Here, we compared galectin-3 distribution in the retina of WT and *mertk*^−/−^ mice at P20 (Mazzoni et al., 2019). At this early age, we observed increased galectin-3 labeling in *mertk*^−/−^retina with a distribution suggestive of Müller glia localization (Figure 2A). Indeed, co-staining of frozen retina sections of the Müller marker protein glutamine synthetase and galectin-3 revealed that the majority of galectin-3 localizes to Müller glia in WT mouse retina (Supplementary Figure S2). Immunoblotting quantification confirmed significantly elevated levels of galectin-3 in the retina of mice lacking MERTK at P20, and levels further increased by P28 (Figures 2B,C). Retinal galectin-3 levels at P35 were the same as levels at P28 (Figures 2B,C).

### Lack of Galectin-3 Exacerbates Retinal Degeneration Due to Inherited MERTK Deficiency That Is Associated Specifically With Early Müller Glia Activation

**Figure 3:**
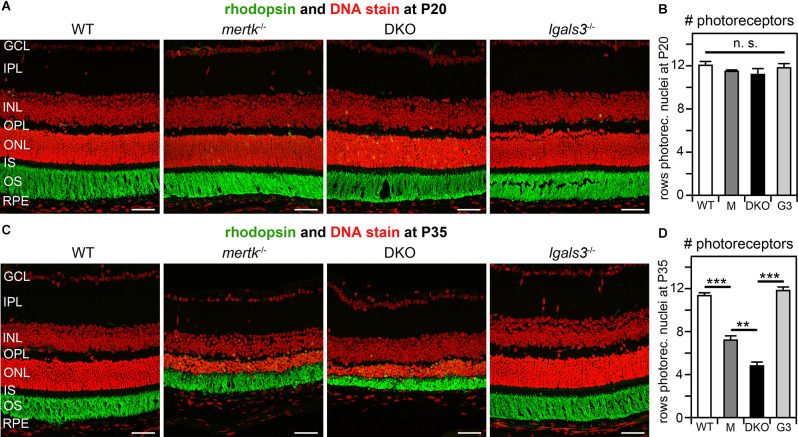
Galectin-3 deficiency in mice lacking MERTK exacerbates photoreceptor degeneration. Dissected eyeballs from WT, *mertk*^−/−^, DKO, and *lgals3*^−/−^ mice at P20 **(A,B)** or P35 **(C,D)** were processed for sectioning and immunofluorescence microscopy. Representative fields in **(A,C)** show photoreceptor outer segment marker rhodopsin (green) and nuclei counterstain (red). Retinal layers are also marked as follows: GCL, ganglion cell layer; IPL, inner plexiform layer; INL, inner nuclear layer; OPL, outer plexiform layer; ONL, outer nuclear layer; IS, photoreceptor inner segments; OS, photoreceptor outer segments; RPE. All scale bars: 40 μm. Bars in **(B,D)** show numbers of rows of photoreceptor nuclei with reduction indicative of photoreceptor degeneration at P35 and counted in images as in **(A,C)**. Bars show mean ± s. d.; *n* = four biological samples from four different mice for each genotype, white bars, WT; dark gray bars, *mertk*^−/−^ (M); black bars, DKO; light gray bars, *lgals3*^−/−^. Data were analyzed by ANOVA; n. s., ANOVA showed no significant difference; no *post-hoc* testing was performed. Select differences established by *post-hoc* testing are indicated, **p < 0.01; ***p < 0.005.

To test whether galectin-3 is functionally relevant for retinal degeneration due to the lack of MERTK, we next backcrossed our existing *mertk*^−/−^ and *lgals3*^−/−^ mice to generate DKO mice lacking both MERTK and galectin-3. Immunoblotting confirmed that DKO tissues lacked both MERTK and galectin-3 proteins (Supplementary Figure S1). Rhodopsin outer segment marker immunofluorescence and cell nuclei microscopy of tissue sections did not reveal phenotypic differences by genotype at P20 (Figure 3A). Moreover, photoreceptor numbers were similar across all genotypes at P20 as indicated by the same number of rows of photoreceptor nuclei in the outer nuclear layer (ONL; Figure 3B). At P35 both *mertk*^−/−^ and DKO retina showed photoreceptor degeneration, while *lgals3*^−/−^ retina did not differ from WT retina (Figures 3C,D). Strikingly, photoreceptor degeneration in DKO retina was more pronounced than degeneration in*mertk*^−/−^ retina at P35 (Figure 3D). We also noticed that both *mertk*^−/−^ and DKO inner retina were thinner than the inner retina of WT and *lgals3*^−/−^ retina at P35 (Figure 3C). While determining the cause of this observation will need experimental follow-up, we speculate that inner retina thickness may be decreased in part due to reduced synapse density thinning plexiform layers.

**Figure 4:**
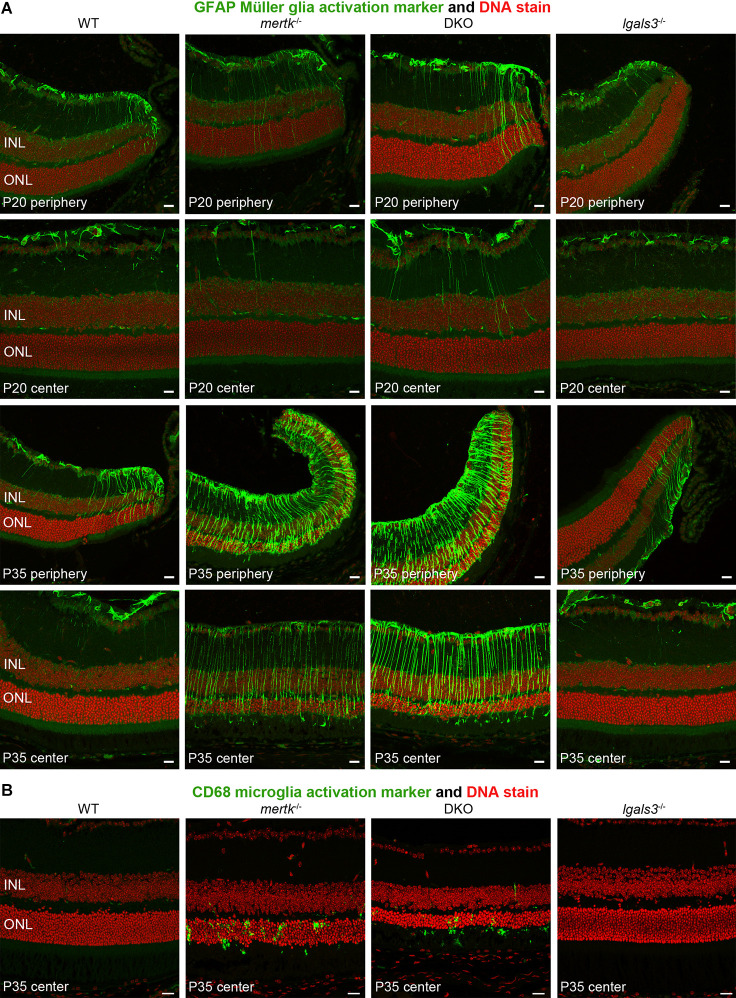
Galectin-3 deficiency in mice lacking MERTK causes enhanced GFAP expression indicative of Müller glia activation. Tissues from age-matched WT, *mertk*^−/−^, DKO, and *lgals3*^−/−^ mice (ages indicated in panels) were processed side-by-side for sectioning and immunofluorescence microscopy of GFAP **(A)** and CD68 **(B)**. **(A)** Images show more pronounced peripheral GFAP (green) at P20 and more pronounced central GFAP in DKO as compared to *mertk*^−/−^ at P35. WT and *lgals3*^−/−^ showed modest GFAP labeling at both ages as expected. **(B)** At P35, *mertk*^−/−^ and DKO retina but not WT and *lgals3*^−/−^ retina showed CD68 labeling in the outer retina, with no apparent difference between *mertk*^−/−^ and DKO. In all fields, nuclei counterstain is shown in red. Inner nuclear layer (INL) and outer nuclear layer (ONL) are indicated to show tissue orientation. Representative images are shown of four eyes from four different mice analyzed per staining, genotype, and age. All scale bars: 40 μm.

**Figure 5:**
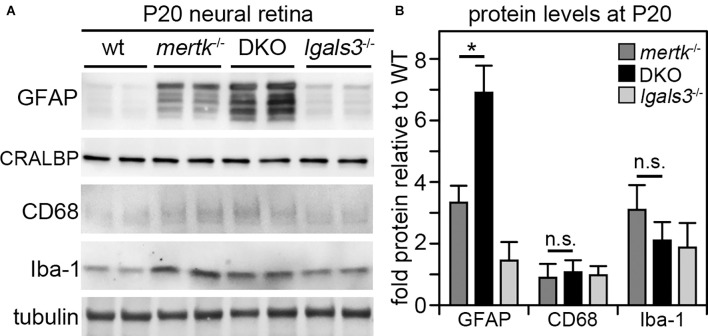
Galectin-3 deficiency in mice lacking MERTK robustly increases GFAP protein levels by P20 implying Müller glia activation at an age prior to photoreceptor degeneration. Tissues from age-matched WT, *mertk*^−/−^, DKO, and *lgals3*^−/−^ mice were dissected to yield isolated neural retina samples, which were analyzed by comparative immunoblotting. Two representative samples from two different mice of each genotype are shown on the same blot in **(A)**. Blots were probed for Müller activation marker GFAP, constitutive Müller glia protein CRALBP, microglia activation marker CD68, and constitutive microglia protein Iba-1 as indicated. Tubulin reprobing was used to control for the total sample load. Quantification of results from blots as in **(A)** yielded bar graph shown in **(B)**. Bars show mean ± s. d.; *n* = four biological samples from four different mice. Data were analyzed by ANOVA; * indicates *p* < 0.05; n. s. indicates no significant difference (*p* > 0.05).

Next, we determined if galectin-3 deficiency affected Müller glia. Indeed, immunofluorescence microscopy showed that GFAP, indicative of Müller glia activation, was elevated in DKO retina as compared to *mertk*^−/−^ retina, with a modest increase apparent in the peripheral retina at P20 and extensive increase in central retina at P35 (Figure 4A). Müller glia in *lgals3*^−/−^ mice did not show GFAP expression above WT at either age tested (Figure 4A). Staining for the microglia activation marker CD68 showed similar numbers and distribution of activated microglia in the outer retina at P35 in both *mertk*^−/−^ and DKO retina (Figure 4B). CD68-positive microglia were absent from WT and *lgals3*^−/−^ retina at P35 (Figure 4B). Of note, we did not see significant CD68 labeling in the outer retina in any strain tested at P20 Supplementary Figure S3. These observations of Müller glia and microglia *in situ* were corroborated and extended by quantification of GFAP and CD68, the Müller and microglia activation markers, respectively. At P20, GFAP showed striking excess activation in the DKO retina, with levels approximately doubled compared to *mertk*^−/−^ retina and a ~7-fold increase compared to levels in WT retina (Figures 5A,B). In contrast, levels of microglia activation were negligible and did not differ between *mertk*^−/−^ and DKO mice (Figures 5A,B). Notably, GFAP and CD68 protein levels in *lgals3*^−/−^ retina were as low as levels in WT retina, demonstrating that galectin-3 deficiency alone does not cause glia activation (Figures 5A,B). Altogether, these results indicate that lack of galectin-3 causes excessive activation of Müller glia prior to microglia activation and leads to more severe retinal degeneration due to MERTK deficiency.

### Lack of Galectin-3 Exacerbates Acutely Induced Retinal Degeneration

**Figure 6:**
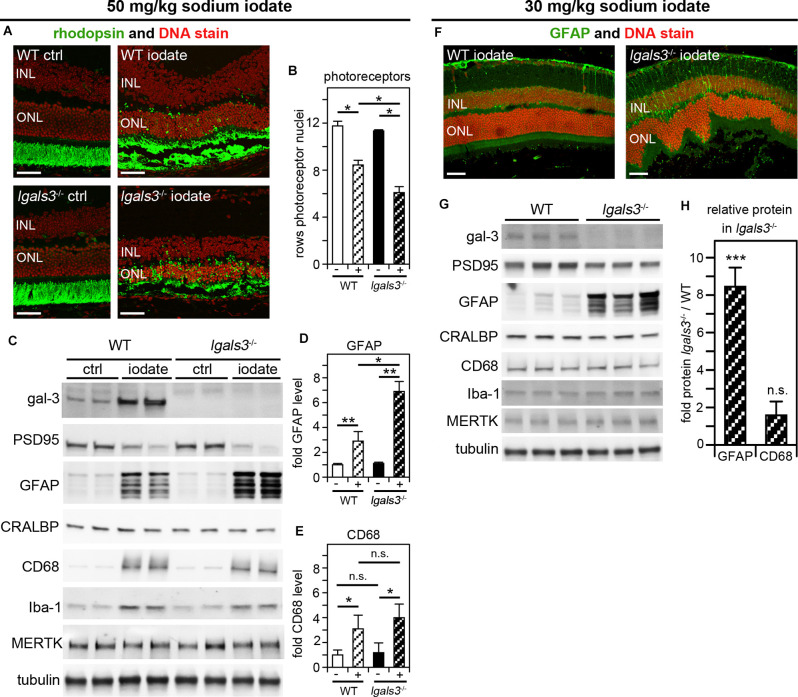
Galectin-3 deficiency increases sensitivity to sodium iodate-induced retinal damage, which involves galectin-3 upregulation in WT retina. At P28, WT and *lgals3*^−/−^ mice received injections of 50 mg/kg sodium iodate or control solvent alone (ctrl) **(AvE)** or 30 mg/kg sodium iodate **(F–H)**. Five days after sodium iodate administration eyeballs were either processed for sectioning and immunofluorescence microscopy **(A,F)** or dissected to yield isolated neural retina samples for immunoblotting **(C,G)**. **(A)** Representative fields show rhodopsin photoreceptor outer segment marker (green) and nuclei counterstain (red). Scale bars: 40 μm. Control treatment did not affect the retina regardless of genotype (panels ctrl). Both iodate-treated WT and *lgals3*^−/−^ retina (panels iodate) showed outer retina disruption. **(B)** Quantification of rows of photoreceptor nuclei revealed more pronounced photoreceptor loss in iodate-treated *lgals3*^−/−^ retina than in WT retina (compare striped bars). Bars compare rows of photoreceptor by genotype and treatment, with white bars indicating WT, black bars indicating *lgals3*^−/−^, and striped bars indicating sodium iodate treatment. Bars show mean ± s. d.; *n* = 3 biological samples from three different mice. **(C)** A representative immunoblot is shown comparing two neural retina samples from two different mice for each genotype and treatment as indicated. Probes as indicated, were applied to test galectin-3 and MERTK expression, PSD95 as a neural marker, as well as Müller and microglia markers as for Figure 5. Tubulin was used as total sample loading control. **(D,E)** Protein levels were quantified from blots as in **(C)** to yield bar graphs. **(D)** GFAP quantification relative to CRALBP, a protein primarily expressed by Müller cells in the neural retina, revealed increased Müller glia activation in *lgals3*^−/−^ mice as compared to WT mice if both received sodium iodate treatment. **(E)** CD68 quantification relative to Iba-1, a microglia marker, showed that microglia activation upon sodium iodate treatment did not differ between WT and *lgals3*^−/−^ mice. For **(B,D,E)** differences among tissues was assessed using ANOVA; * indicates *p* < 0.05; n. s. indicates no significant difference (*p* > 0.05). **(F)** Following low dose sodium iodate treatment, representative immunofluorescence microscopy fields show obvious GFAP Müller activation marker and outer retina disruption only in *lgals3*^−/−^ retina. Nuclei are counterstained in red. Scale bars: 40 μm. Panel **(G)** shows immunoblot membrane comparison of photoreceptor, Müller, and microglia marker proteins (as in **B**) from three neural retina samples from three different mice for each treatment. Quantification of relative protein levels in *lgals3*^−/−^ neural retina compared to WT neural revealed a significant increase in GFAP—but not in CD68—indicating specifically Müller glia activation in mice lacking galectin-3 treated with low dose sodium iodate. **(H)** Bars show mean ± s. d.; *n* = 3 biological samples from three different mice. The Student’s t-test was used to establish differences between *lgals3*^−/−^ and WT protein content; ** indicates *p* < 0.01; *** indicates *p* < 0.005; n. s. indicates no significant difference (*p* > 0.05).

We wondered if galectin-3 was relevant specifically for inherited retinal degeneration due to MERTK deficiency. To test this directly, we selected to investigate a model of acutely induced retinal degeneration caused by sodium iodate application. In this well-established model, systemic sodium iodate acts dose-dependently to cause cytotoxic oxidative damage to RPE cells, resulting in swift RPE and outer retina degeneration (Chowers et al., 2017). Here, we first applied sodium iodate at 50 mg/kg to age- and strain-matched WT and *lgals3*^−/−^ mice and analyzed the effects 5 days later. Figure 6A shows that both WT and *lgals3*^−/−^ retina demonstrate severe retinal damage, although outer segment rhodopsin appeared especially disrupted in *lgals3*^−/−^ retina. Indeed, sodium iodate reduced the number of photoreceptors in both WT and *lgals3*^−/−^ mice compared to control mice of the same genotype, but a reduction in *lgals3*^−/−^ mice was significantly more pronounced than the reduction in WT mice (Figure 6B). Moreover, we found that the sodium iodate-induced increase in GFAP was significantly higher in *lgals3*^−/−^ than in WT retina by more than two-fold (Figures 6C,D). In contrast, microglia activation did not differ between mice with and without galectin-3 (Figures 6C,E). Finally, we adjusted the dose of sodium iodate to just below levels that are toxic to WT retina. 5 days after administering 30 mg/kg sodium iodate, WT retina had normal morphology, while *lgals3*^−/−^ retina exhibited distortion of the outer retina (Figure 6F, compare WT and *lgals3*^−/−^ appearance of DNA stain) and increased GFAP immunolabeling (Figure 6F, compare WT and *lgals3*^−/−^ GFAP stain). Indeed, under these treatment conditions, levels of Müller activation marker GFAP in *lgals3*^−/−^ retina were ~7.5-fold greater than in WT retina (Figures 6G,H). Microglia activation was negligible and did not differ by genotype (Figures 6G,H). These data demonstrate that lack of galectin-3 robustly exacerbates Müller glia activation and worsens tissue damage in acutely induced retinal injury. Altogether, our results from two distinct models of retinal degeneration support a supportive role for galectin-3 in Müller glia modulation that serves to protect the retina in disease.

## Discussion

Müller glia are essential for the development of neuronal synapses and long-term homeostasis of the healthy retina. Moreover, their activation is associated with diverse forms of retinal disease (Reichenbach and Bringmann, 2013). Recently, Müller glia have also been shown to play an important role in clearance phagocytosis of cellular debris during both retinal development and retinal injury (Bejarano-Escobar et al., 2017; Sakami et al., 2019; Nomura-Komoike et al., 2020). Here, we set out to investigate their molecular mechanism, exploring immortalized and primary Müller cell culture models in quantitative *in vitro* phagocytosis assays. We found that like RPE cells, Müller glia express and employ the TAM engulfment receptor MERTK for clearance phagocytosis. However, Müller cells utilize extracellular protein S and galectin-3 as MERTK ligands indistinguishably: both proteins, if added at the time of particle challenge, promote phagocytosis with similar efficiency, and their effects are additive if provided at low concentrations and saturated if provided at high concentrations. We found previously that protein S—but not galectin-3—promotes clearance phagocytosis by RPE cells in culture. Our experiments on Müller glia (reported in this study) and RPE (reported in Esposito et al., 2021) differ in cell type alone; in both sets of experiments, we used the same extracellular ligand preparations and the same purified, phosphatidylserine-positive POS. We do not have an explanation for this finding at this time. We speculate that the extracellular ligand-binding domain of MERTK may differ structurally between the two cell types, accommodating galectin-3 on Müller glia but not on RPE. Future studies could test chimeric ligands combining protein S and galectin-3 domains to determine essential motifs for both types of phagocytosis.

We have not seen other reports directly investigating Müller glia phagocytosis using quantitative *in vitro* assays probing Müller cells in culture. While cell culture assays do not fully mimic *in vivo* phagocytosis and thus must be interpreted with caution, they have proven powerful in identifying essential aspects of phagocytic machineries in other cell types. The experiments reported here illustrate that these *in vitro* assays offer the opportunity to directly compare different primary cell types under otherwise identical conditions. Comparing cultured human, WT mouse, and mutant mouse Müller glia to each other and to primary RPE should yield further mechanistic insight in the future.

The intriguing results from our cell culture assays served as a starting point for us to probe the function of galectin-3 in Müller glia *in vivo*. We found excess protein levels of galectin-3 in Müller cells in the *mertk*^−/−^ model of retinal degeneration at an age prior to detectable photoreceptor distress or any retinal abnormality, let alone overt retinal degeneration. To date, we do not know the purpose of Müller glia elevating galectin-3, and we will follow up on this observation in future studies. However, our findings suggest that elevation of galectin-3 is not a secondary phenomenon of late-stage disease. Moreover, DKO mice lacking both galectin-3 and MERTK show significantly more pronounced Müller glia activation and retinal degeneration compared to mice lacking MERTK alone. Our experiments comparing WT and *lgals3*^−/−^ mice in the sodium iodate model of acute injury confirm that lack of galectin-3 causes more severe Müller glia activation and retinal degeneration. Given the lack of microglia activation in response to low dose sodium iodate, our results suggest a microglia-independent role of galectin-3 in restraining activation of Müller cells. As Müller glia activation occurs at P20 prior to obvious photoreceptor degeneration, Müller cell activation may have a direct role in stimulating photoreceptor distress or death, rather than occurring secondary to photoreceptor damage due to long-established RPE phagocytic defects. However, we cannot exclude the possibility that early RPE cell responses in MERTK deficiency or upon sodium iodate injury prior to noticeable photoreceptor degeneration affect Müller cells. While this intriguing possibility requires further investigation, the data presented here match and extend our previous findings of activated inflammatory pathways and microglia in MERTK-deficient Royal College of Surgeons (RCS) rats prior to the age at which MERTK begins to function in diurnal phagocytosis of the RPE in WT rats (Lew et al., 2020). However, we find here that DKO mice do not differ in microglia activation, indicating a Müller cell-specific role of galectin-3. Similarly, WT and *lgals3*^−/−^ microglia activation is similar in response to sodium iodate at a level that is toxic to the WT retina. Most notably, levels of sodium iodate that are not overtly toxic to WT retina yield obvious Müller activation and retinal distress in mice lacking galectin-3, while very modest background level microglia activity is the same regardless of whether or not galectin-3 is present. Altogether, lack of galectin-3 causes overactivation primarily of Müller glia and worsens retinal injury, and this is not specific to retinal degeneration due to loss of the galectin-3 receptor MERTK. This is somewhat surprising, as galectin-3 is well-documented to modulate microglia activation in the brain in a variety of diseases (reviewed by Rahimian et al., 2018). Incubating LPS with isolated neural retina *ex vivo* yields galectin-3 in Müller cells and microglia (Bauer et al., 2016). Experimental optic nerve crush-induced or diabetes-associated damage to retinal ganglion cell axons *in vivo* is less severe in mice lacking galectin-3, and this is associated with reduced microglia/macrophage activity (Abreu et al., 2017; Mendonca et al., 2018). Similarly, galectin-3 inhibition in experimental uveitis may reduce disease severity (Liu et al., 2022). Altogether, these earlier studies demonstrate a role for galectin-3 in retinal microglia that worsens the outcome of retinal injury, although there was little focus on contributions of galectin-3 in Müller glia. As galectin-3 may exert opposite effects on microglia and Müller glia in the context of a retinal disease process, therapies manipulating galectin-3 may need to consider cell type-specific targeting. Of note, in our experimental models, we studied retinal distress at the very onset and found primarily Müller glia activation prior to microglia involvement. However, extensive cross-talk of microglia and Müller cells will further complicate disease mechanisms in any retinal disease as it progresses (Wang and Wong, 2014; Abcouwer, 2017; Portillo et al., 2017; Di Pierdomenico et al., 2020).

While galectin-3 has not previously been studied in Müller cells, Koh and colleagues recently reported decreased levels of glutamine synthetase, a key metabolic enzyme of Müller glia, in the retina of RCS rats lacking MERTK and in mice lacking MERTK only in Müller cells (Koh et al., 2018). In addition, MERTK loss from Muller glia resulted in Müller cell activation (Koh et al., 2018). Our results add to the concept that Müller glia may be impaired by MERTK mutation directly, cell-autonomously, and independently of RPE dysfunction.

Our experiments identify extracellular galectin-3 as Müller cell phagocytic ligand for MERTK. However, in the MERTK-deficient retina, any effect of galectin-3 would surely be independent of MERTK. Given the plethora of cellular activities and protein-protein interactions in which galectin-3 may be involved, we cannot speculate on the specific MERTK-independent nature of galectin-3 function in Müller glia. Future studies are needed to characterize this function.

Together with earlier work, our studies illustrate that there are important differences in molecular pathways employed by the three major professionally phagocytic cell types of the retina. While galectin-3 is expressed by all of them, we found no role for galectin-3 in routine, MERTK-dependent RPE phagocytosis, despite its function as a phagocytic ligand for MERTK in Müller glia. Lack of galectin-3 exacerbates Müller activation, aggravating photoreceptor degeneration in the two models we studied, while microglia in galectin-3-defective models have been shown to be less active and damaging especially to retinal ganglion cells. Each cell type, therefore, requires specific, focused investigation to identify molecular mechanisms. Moreover, loss of function or inhibition of molecules may exert differential effects on these different cell types, complicating outcomes for the retina as a whole. This will be an important consideration for future work as well as for the design of retinal therapies.

## Data Availability Statement

The raw data supporting the conclusions of this article will be made available by the authors, without undue reservation.

## Ethics Statement

The animal study was reviewed and approved by the Institutional Animal Care and Use Committee of Fordham University.

## Author Contributions

This study was designed by DL and SF. DL, MM, and SF performed experiments, analyzed and interpreted results. DL, MM, and SF wrote the manuscript. All authors contributed to the article and approved the submitted version.

## Conflict of Interest

The authors declare that the research was conducted in the absence of any commercial or financial relationships that could be construed as a potential conflict of interest.

## Publisher’s Note

All claims expressed in this article are solely those of the authors and do not necessarily represent those of their affiliated organizations, or those of the publisher, the editors and the reviewers. Any product that may be evaluated in this article, or claim that may be made by its manufacturer, is not guaranteed or endorsed by the publisher.
