# Supplementary material for: Galectin-3 Promotes Müller Glia Clearance Phagocytosis via MERTK and Reduces Harmful Müller Glia Activation in Inherited and Induced Retinal Degeneration
Source: Front Cell Neurosci. 2022 May 31;16:878260. doi: 10.3389/fncel.2022.878260 (PMC9194531; doi:10.3389/fncel.2022.878260)
Supplement: Supplementary file 1 [file Data_Sheet_1.PDF]

Supplementary Material

Lew et al.

Galectin-3 promotes Müller glia clearance phagocytosis via MERTK and acts as a neuroprotectant to reduce Müller gliosis in inherited and induced retinal degeneration

Supplementary Table 1. Antibodies used

**target protein**	**application**	**dilution used**	**catalog #, supplier**
α-tubulin	WB	1:2000	9099, Cell Signaling, Danvers, MA
Iba-1	IF	1:500	019-19741, Fujifilm Wako Chemicals,
Iba-1	WB	1:2000	016-20001, Fujifilm Wako Chemicals
CD68	IF	1:500	MCA1957, Biorad, Hercules, CA
CD68	WB	1:2000	9777, Cell Signaling
CRALBP	WB	1:1000	NB100-74392, Novus Biologicals, Littleton, CO
galectin-3	IF	1:50	AF1197, R & D Systems, Minneapolis, MN
galectin-3	WB	1:500	14979-1-AP, Proteintech, Rosemont, IL
Glutamine synthetase	IF	1:1000	MAB302, MilliporeSigma
GFAP	IF/WB	1:500/1:5000	MAB360, MilliporeSigma
MERTK	WB	1:1000	270448, Abcam, Waltham, MA
PSD95	WB	1:5000	MAB1598, MilliporeSigma
rhodopsin (B6-30)	IF and WB	1:250/ 1:500	NBP2-25160, Novus Biologicals

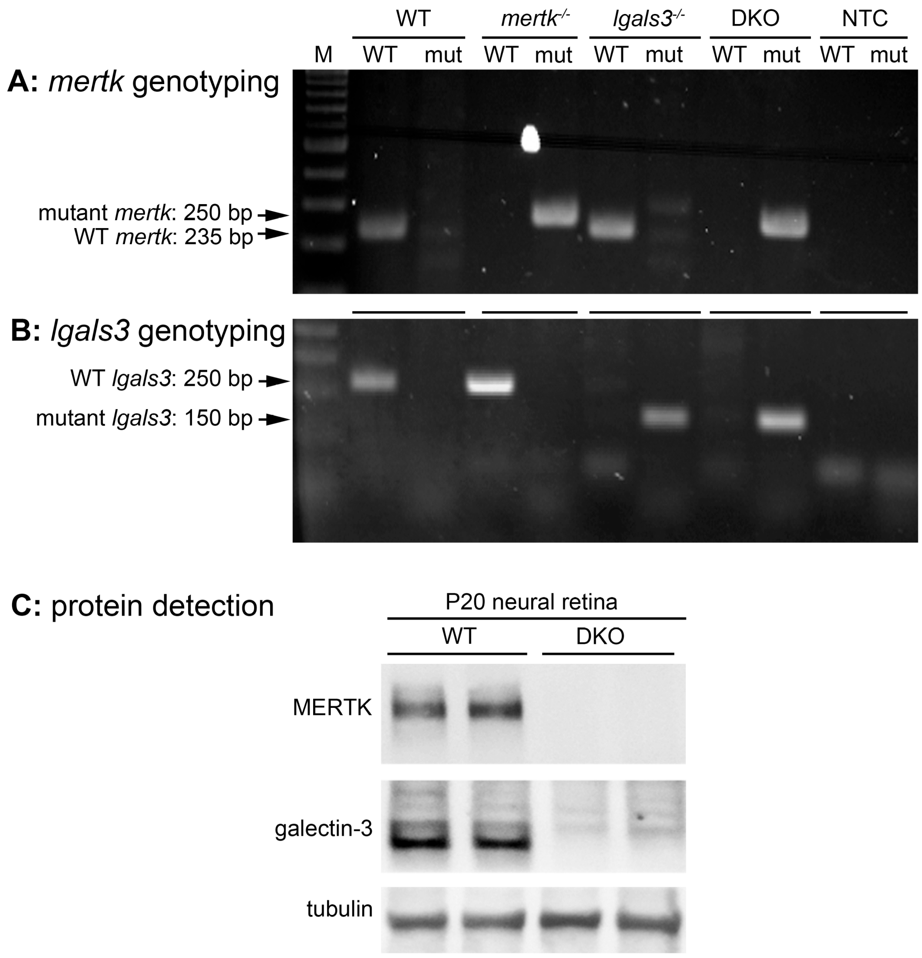

Supplementary Figure S1. Genomic PCR and immunoblotting confirmed *mertk*$^{-/-}$ / *lgals3*$^{-/-}$ genotype and lack of MERTK and galectin-3 protein expression, respectively, of DKO mice. DNA extracted from tail clips of P15-20 mice of genotypes as indicated using the DirectPCR Lysis Reagent for mouse tails (Viagen Biotech) according to the manufacturer's instructions were used as template for standard genomic PCRs using the BlueChoice Taq system (Denville Scientific, Denville, NJ). WT and mutant alleles of MERTK **(A)** and lgals3 **(B)** were amplified in separate reactions and applied in alternate labels on gels (WT, mut, as indicated). Dissected neural retina was subjected to SDS-PAGE and immunoblotting as described in Methods. Panels in (**C**) show a representative immunoblot probed for MERTK, galectin-3, and tubulin as loading control. Two retinas from 2 different WT and DKO mice, as indicated, were analyzed side-by-side. No specific bands were obtained for MERTK or galectin-3 in DKO tissues. NTC, no template control.

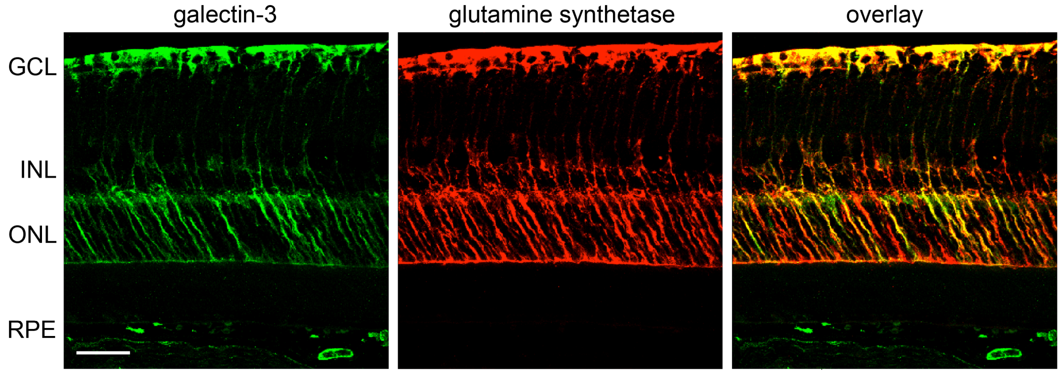

Supplementary Figure S2. Galectin-3 localizes to Müller cells in wild-type mouse retina. The image shows a cryosection of a 2 month-old wild-type mouse stained for galectin-3 (green) and glutamine synthetase (red), a Müller cell marker protein. Scale bar: 40 µm. A representative maximal projection is shown of eyes from 3 mice analyzed. The overlay field on the right shows that most galectin-3 labeling colocalizes with the Müller cell marker. Of note, Müller cell levels of galectin-3 show some variability.

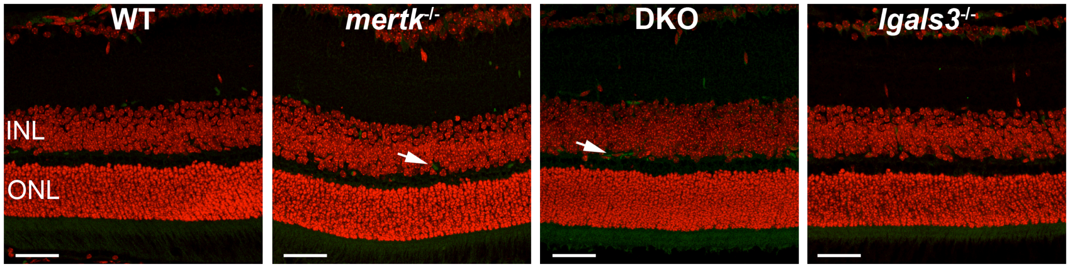

Supplementary Figure S3. DKO and *mertk*$^{-/-}$ retina at P20 do not differ in levels and localization of microglia activation marker CD68. Tissues from P20 WT, *mertk*$^{-/-}$, DKO, and *lgals3*$^{-/-}$ mice (as indicated in panels) were processed side-by-side for sectioning and immunofluorescence microscopy of CD68. Representative images show no CD68-labeled cells in WT and *lgals3*$^{-/-}$ retina. *mertk*$^{-/-}$ and DKO show similar levels of small numbers of CD68-positive cells in the inner retina only (examples indicated by arrows). In all fields nuclei counterstain is shown in red. Inner nuclear layer (INL) and outer nuclear layer (ONL) are indicated to show tissue orientation. 4 eyes from 4 different mice were analyzed per genotype. Scale bars: 40 µm.
